# Recruitment of C4b-binding protein is not a complement evasion strategy employed by *Staphylococcus aureus*

**DOI:** 10.1099/mic.0.001391

**Published:** 2023-09-05

**Authors:** Shuxian Li, Serena Bettoni, Frida Mohlin, Joan A. Geoghegan, Anna M. Blom, Maisem Laabei

**Affiliations:** ^1^​ Department of Life Sciences, University of Bath, Bath, BA2 7AY, UK; ^2^​ Division of Medical Protein Chemistry, Department of Translational Medicine, Lund University, Malmö, Sweden; ^3^​ Institute of Microbiology and Infection, University of Birmingham, Edgbaston, Birmingham, B15 2TT, UK

**Keywords:** C4b-binding protein, complement, immune evasion, *Staphylococcus aureus*

## Abstract

Complement offers a first line of defence against infection through the opsonization of microbial pathogens, recruitment of professional phagocytes to the infection site and the coordination of inflammatory responses required for the resolution of infection. *Staphylococcus aureus* is a successful pathogen that has developed multiple mechanisms to thwart host immune responses. Understanding the precise strategies employed by *S. aureus* to bypass host immunity will be paramount for the development of vaccines and or immunotherapies designed to prevent or limit infection. To gain a better insight into the specific immune evasion mechanisms used by *S. aureus* we examined the pathogen’s interaction with the soluble complement inhibitor, C4b-binding protein (C4BP). Previous studies indicated that *S. aureus* recruits C4BP using a specific cell-wall-anchored surface protein and that bound C4BP limits complement deposition on the staphylococcal surface. Using flow-cytometric-based bacterial-protein binding assays we observed no interaction between *S. aureus* and C4BP. Moreover, we offer a precautionary warning that C4BP isolated from plasma can be co-purified with minute quantities of human IgG, which can distort binding analysis between *S. aureus* and human-derived proteins. Combined our data indicates that recruitment of C4BP is not a complement evasion strategy employed by *S. aureus *.

## Introduction

*Staphylococcus aureus* exists as a bacterial commensal of the human nares and skin with significant pathogenic potential, frequently causing a suite of diseases ranging from skin and soft tissue infections to life-threatening bacteraemia [1]. Treatment of *S. aureus* has been made complicated by the worldwide emergence of multiple lineages that have acquired resistance to a multitude of antibiotics [2]. Consequently, the WHO has listed *S. aureus* as a ‘priority pathogen’, where the development of novel antimicrobials and immunotherapeutics are urgently required to treat infections [3]. To develop effective therapeutic interventions, a greater understanding of the pathophysiological process of *S. aureus* disease is central.

An immediate host response to *S. aureus* infection is governed by complement, a sophisticated evolutionary conserved system that promotes the detection of microbes and mediates the coordination of inflammatory processes that serve to eliminate foreign species [4]. The complement system consists of multiple soluble and membrane-bound proteins with specific activator and inhibitor functions. Complement is activated by microbes via three distinct pathways (the classical, lectin and alternative pathways), which join at the level of C3 convertase formation and subsequent cleavage of the central C3 molecule. C3 is cleaved into C3a, an inflammatory modulator component and C3b, the major opsonin that can deposit onto cellular surfaces and promote the uptake of microbes by professional phagocytes. Extended C3b deposition results in the formation of the C5 convertases, which cleave C5 into C5a, a potent anaphylatoxin and C5b, a central initiator of the membrane attack complex (MAC).

To limit damage generated by complement activation, the host has evolved several fluid-phase and immobilized proteins that act as complement inhibitors [5]. C4b-binding protein (C4BP), a large 500 kDa multimeric glycoprotein and Factor H (FH), a 155 kDa serum glycoprotein are two soluble complement inhibitors. C4BP limits classical and lectin complement pathways through its ability to bind and restrict the function of activated complement C4b. C4BP can act as a cofactor to the serine protease Factor I (FI), directing the inactivation of soluble and membrane bound C4b, inhibiting the formation of the classical C3 convertase. In addition, C4BP accelerates the natural decay of the classical C3 convertase and can serve as a cofactor to FI in the cleavage of fluid-phase C3b, thus interfering with the alternative complement pathway [6]. FH represents the master regulator of the alternative pathway; FH acts as a cofactor for FI-mediated C3b cleavage, competes with Factor B for interaction with C3b, preventing the formation of fluid-phase C3 convertase and functions to promote the dissociation of the alternative C3 convertase [7].

Microbes have evolved multiple strategies to evade the complement system, promoting host colonization and access to resources [8]. One strategy used by several bacteria is the recruitment of host complement inhibitors, such as C4BP, to the microbial surface, limiting complement activation and subsequent eradication [6, 8]. Previous work has indicated that *S. aureus* recruits C4BP via the cell-wall-anchored serine-aspartate repeat (Sdr) protein E (SdrE) and bone sialoprotein binding protein (Bbp), an allelic variant of SdrE with 75 % sequence identity [9], resulting in C4BP being localized to the bacterial surface, disrupting complement activation [10, 11]. The distribution of the *sdrE/bbp* gene has been examined previously; Sabat *et al*. reported that the *sdrE* gene was present in 89.5 % (445/497) of strains from a genetically diverse, clinical collection (MSSA *n*=382; MRSA *n*=115) where *sdrE* distribution did not differ between MSSA and MRSA isolates [12]. Another study investigated the carriage of the *sdr* locus among diverse *S. aureus* lineages revealing that the *sdrE* gene was present in 68.1 % (196/288) isolates [13]. The SdrE sequence exhibits significant variation between different *S. aureus* lineages compared to within lineages, with higher levels of variation observed in the protein domains at the host interface [14].

One aim of this study was to determine if additional methods of C4BP recruitment existed and to investigate to what degree C4BP recruitment is conserved across genetically distinct *S. aureus* isolates. Understanding the conservation and existence of alternative staphylococcal C4BP binding proteins is important to enhance our understanding of *S. aureus* immune evasion and to direct future therapeutic intervention strategies.

Employing flow-cytometry-based, bacteria-protein binding assays and defined *S. aureus* isogenic mutants, we conclude that *S. aureus* does not recruit C4BP under standard laboratory conditions. Our data also provides a precautionary warning that minute quantities of human IgG can be associated with C4BP following purification from human plasma, which can distort results, particularly if bacteria under question strongly bind IgG.

## Methods

### Bacterial isolates and growth conditions

*S. aureus* strains were grown in tryptic soy broth (TSB) at 37 °C with shaking (180 r.p.m.). *S. aureus* transposon mutants were obtained from the Nebraska Transposon Mutant Library [15] and were grown in TSB containing erythromycin (5 µg ml^−1^). Newman *sdrE:*:Tn and Newman *spa*::Tn were generated through transduction using ϕ11. Transductants containing the inserted transposon were examined on tryptic soy agar containing erythromycin (10 µg ml^−1^). SdrE and Spa transposon mutants were verified for correct Tn insertion by colony PCR using gene specific primers; sdrE_FW: 5′-CCAACTACACCTCAAGAATCTAC-3′; sdrE_RV: 5′-GGCTTGTTTCTTTACCTGCTG-3′ and spa_FW: 5′-CCTCAGCACATTCAAAGCC-3′; spa_RV: 5′-AGCCGTTACGTTGTTCTTC-3′. *Lactococcus lactis* MG1363 was used to express *S. aureus* proteins, protein A and SdrE, the genes of which had been previously cloned into the expression vector pKS80 [16]. *L. lactis* strains were grown in M17 broth containing 1 % glucose and erythromycin (5 µg ml^−1^) at 30 °C without shaking. *Streptococcus pyogenes* strain AP1 was grown in Todd–Hewitt broth at 37 °C with 5 % CO_2_ without shaking. *Moraxella catarrhalis* strain RH4 was cultured on chocolate agar plates for 24 h at 37 °C with 5 % CO_2_.

### Human serum preparation, C4BP protein purification and labelling

Normal human serum (NHS) was harvested from freshly obtained blood from eight healthy volunteers using vacutainer serum CAT collection tubes (BD). Blood samples were allowed to clot at room temperature for 30 min and then incubated on ice for 1 h. After two rounds of centrifugations at 800 ***g*** at 4 °C for 8 min, serum samples were collected and pooled at 4 °C and then immediately aliquoted and stored at −80 °C. Healthy volunteers provided written informed consent according to the recommendations of the University of Bath, Research Ethics Approval Committee for Health (reference: EP 18/19 108). Heat-inactivated serum (HI-S) was prepared by heating serum at 56 °C for 30 min using a water bath.

C4BP was purified from pooled plasma using barium chloride, anion exchange chromatography and gel filtration as described previously [17]. In addition C4BP was expressed from the pcDNA3 vector in a human kidney 293 cell line as outlined by Blom *et al*. [18]. Lastly C4BP was also purchased (Complement Technologies; A109), aliquoted and stored at −80 °C until use. C4BP was labelled with Dylight 488 using DyLight 488 NHS Ester (ThermoFisher) according to the manufacturer’s instructions. Labelled C4BP were separated from uncoupled dye via buffer exchange using Zeba Spin Desalting Columns (ThermoFisher). The degree of labelling was determined following the manufacturer's instructions. Briefly, for each C4BP preparation, absorbance at 280 nm and at 493 nm was measured by spectrometry, and used in the following calculation:

$$moles\;dye\;per\;mole\;protein=\frac{A\_max\;of\;the\;labelled\;protein\;x\;dilution\;factor\;}{\varepsilon \_fluor\;x\;protein\;concentration\;\left(M\right)}$$

Plasma purified C4BP (4.33), recombinantly produced C4BP (3.74) and C4BP purchased from CompTech (4.36), exhibited similar labelling degrees defined as moles dye per mole protein using this protocol. Labelled C4BP was eluted in PBS and stored at 4 °C. IgM/IgG depleted serum was purchased from Pel-Freez Biologicals, aliquoted and stored at −80 °C until required. Human IgG contamination of C4BP was measured by SDS-PAGE and western blot. Purified C4BP was loaded at 1 and 2 µg and subjected to electrophoresis using a 5 % SDS gel under non-reducing conditions. The gel was transferred onto a PVDF membrane using a Trans blot turbo transfer system (BioRad). The membrane was blocked overnight in TBST containing 5 % semi skinned milk before being incubated with goat anti-human IgG-HRP conjugate (1 : 1000; Life Technologies) for 45 min at room temperature before being visualised using an Amersham-ECL kit (Cytiva).

### Examining bacterial interaction with C4b-binding protein

For *S. aureus *, overnight cultures were 1 : 200 diluted in fresh TSB and sub-cultured to OD_600_=0.5–0.6. For *S. pyogenes *, overnight culture was normalized to OD_600_=0.1 and sub-cultured to OD_600_=0.3–0.4 in Todd–Hewitt broth. For *M. catarrhalis *, bacteria were cultured on chocolate agar plates overnight and streaked onto new chocolate agar plates grown for 7 h. *M. catarrhalis* were scraped from plates and resuspended into 25 % BHI with glycerol. Glycerol stocks were kept in −80 °C and thawed at 37 °C for 30 min before use. For *L. lactis* expressing *S. aureus* surface proteins, bacteria were normalized from stationary phase culture.

Bacteria were harvested via centrifugation at 13 000 ***g*** for 5 min following PBS wash. *S. aureus, S. pyogenes* and *M. catarrhalis* were normalized to OD_600_=2 in PBS. Bacteria were stained using 2 µM Cell Trace Far Red (ThermoFisher) for 20 min at 37 °C with shaking. Unbound dye was removed by washing cells in 1 % BSA/PBS. Bacteria were resuspended in either PBS for labelled C4BP binding experiments, or in GVB^++^ for binding of C4BP from NHS. *L. lactis* were normalized to OD_600_=1 in PBS and stained using 1 µM of Cell Trace Far Red and prepared as above.

To detect bacterial binding of labelled C4BP, 50 µl stained bacteria cells were mixed with 50 µl labelled C4BP in 96-well plates at 37 °C for 30 min. Bacteria were centrifuged and washed once in 1 % BSA/PBS and resuspended in 100 µl PBS. Bacteria incubated without labelled C4BP was used as negative control. In IgG-C4BP competition experiments, bacteria were pre-incubated with purified human IgG (Sigma) for 30 min, washed three times and then incubated with labelled C4BP for 30 min. Following incubation, bacteria were washed once in 1 % BSA/PBS and resuspended in 100 µl PBS.

To detect binding of C4BP from NHS, 50 µl stained bacteria cells were mixed with 50 µl NHS in 96-well plates at 37 °C for 30 min. Bacteria were centrifuged and washed once in 1 % BSA/PBS. F(ab’)2 monoclonal mouse anti-human C4BP MK104 [19] antibodies were prepared using the F(ab’)2 preparation kit (Pierce). Bacteria were resuspended in 100 µl of F(ab’)2 MK104 in 1 % BSA/PBS (4 µg ml^−1^) for 45 min at room temperature followed by centrifugation and washing 1 × in 1 % BSA/PBS. Bacteria were then stained with goat anti-mouse AF-488 (1 : 1000; Invitrogen) or goat anti-mouse PE (1;1000; Abcam) secondary antibody using the above conditions. Bacteria incubated in the absence of serum was used as a negative control.

Bound C4BP was assessed using FACS CANTO (BD) flow cytometer using wavelength 488 and 633 nm. Stained and unstained bacteria were employed for accurate gating of bacteria; a minimum of 20 000 events were used in all experiments. Data were analysed by FlowJo v10 software (BD Life Sciences).

### Complement C9 deposition analysis

*S. aureus* and *S. pyogenes* were prepared as above for C4BP interaction. Bacteria were incubated with 8 % NHS and increasing concentrations of IgG-free C4BP diluted in GVB^++^ for 1 h at 37 °C. Bacteria were centrifuged and washed once in 1 % BSA/PBS and resuspended in 100 µl FcR blocking reagent (1 : 5, miltenyibiotec). Plates were incubated at 4 °C for 30 min. After washing with 1 % BSA/PBS, bacteria were stained with goat anti-human C9 (Complement Technologies) at 4 °C for 30 min and washing once with 1 % BSA/PBS. Bacteria were when stained with rabbit-anti-goat AF-488 secondary antibody (1 : 1000, Invitrogen) at 4 °C for 30 min. Bacteria incubated in HI-S were used as negative control. C9 deposition was examined as described above for C4BP detection using flow cytometry. The fluorescent intensity for each sample was normalized using NHS without added C4BP as the maximum value and HI-S as the minimum value.

### Statistical analysis

A one-way or two-way ANOVA (GraphPad Prism 9.4.1) was used to examine differences between experimental results where a *P* value<0. 05 was considered to be significant.

## Results

### Interaction of plasma purified C4BP with *S. aureus* and *L. lactis* expressing SdrE

**Fig. 1.**
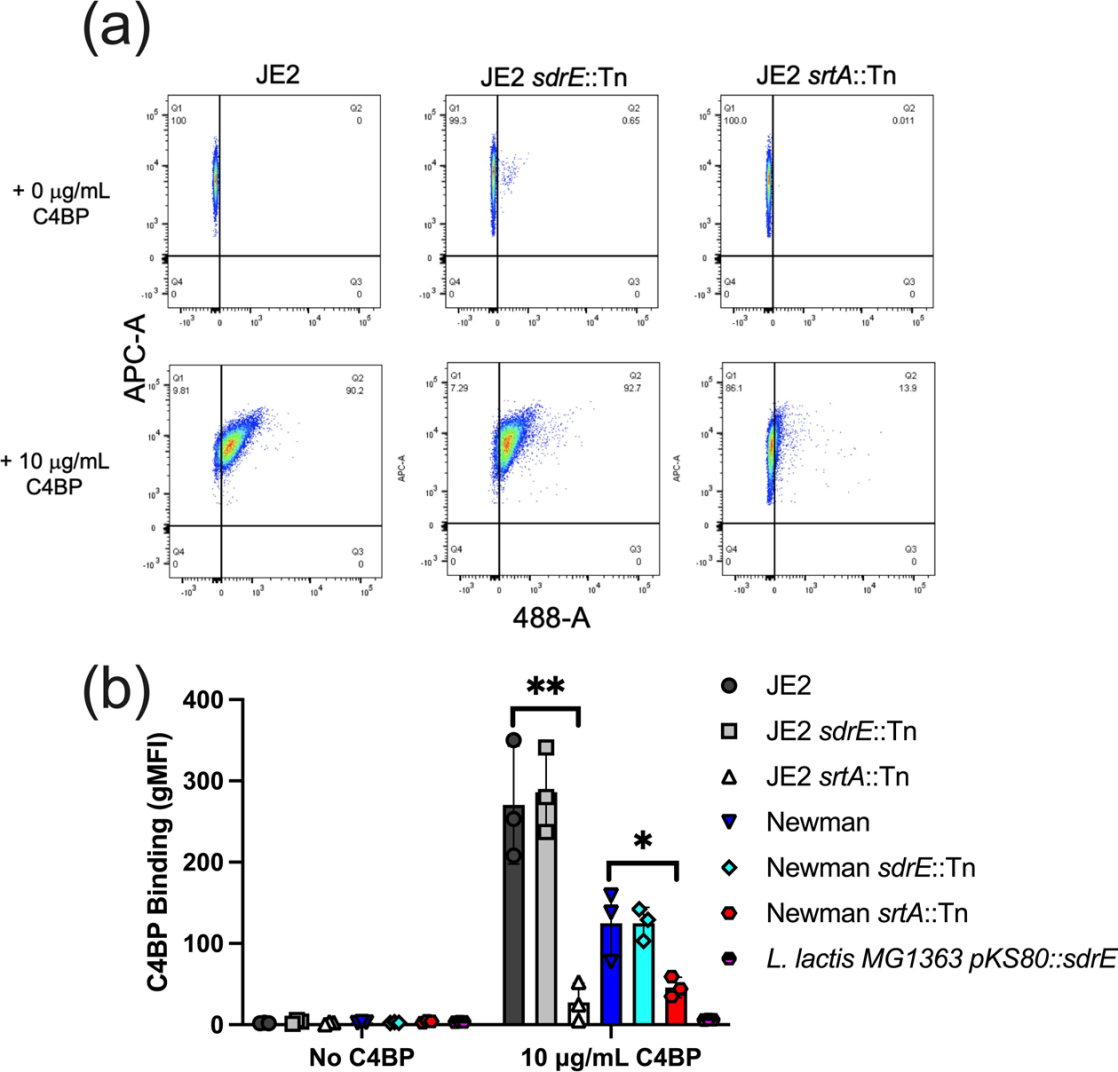
Interaction of plasma purified C4BP with *S. aureus* and *L. lactis* expressing SdrE. Dy488-labelled plasma purified C4BP (10 µg ml^−1^) was incubated with *S. aureus* strains and *L. lactis* expressing SdrE. Bacterial strains were labelled using cell trace Far Red (APC-A). C4BP binding was analysed by measuring the geometric mean fluorescence intensity (gMFI) using a BD FACS Canto flow cytometer. Bars indicate the mean; data points represent three biological replicates and error bars inform the standard deviation. Statistical differences were calculated using a one-way ANOVA analysis using Dunnett’s multiple comparisons test comparing mutants to respective isogenic WT control. *P<0.05, **P<0.01,.

Our original aim was to test whether SdrE was the sole staphylococcal protein that mediated C4BP recruitment to the bacterial surface. To test this, we developed a simple *S. aureus* – C4BP binding assay using DyLight 488-fluorescently labelled plasma purified C4BP (Fig. S1, available in the online version of this article), Cell-trace far red labelled *S. aureus* and flow cytometry. We tested binding of plasma purified C4BP using two commonly used *S. aureus* strains, JE2 and Newman, both of which showed binding of C4BP (10 µg ml^−1^) (Fig. 1a-b). Next, we tested whether inactivation of SdrE, the only described *S. aureus* C4BP binding protein [11], impacted C4BP recruitment. Surprisingly we saw no difference in binding of C4BP between WT and *sdrE*::Tn mutants. Interestingly, when we overexpressed the gene coding for SdrE in the heterologous host, *Lactococcus lactis *, we did not observe any C4BP binding (Fig. 1b). We confirmed that *L. lactis* was expressing SdrE following extraction of cell-wall proteins and Western blotting using anti-SdrE serum [16] (Fig. S2A). Lastly, we tested the importance of a functional sortase A gene in the recruitment of C4BP. Sortase A is a central enzyme in the covalent linkage of staphylococcal cell-wall-anchored (CWA) proteins to peptidoglycan [20]. Here we observed significantly less C4BP binding in both JE2 and Newman generated *srtA* mutants (Fig. 1b), indicating the importance of CWA proteins for plasma purified C4BP interaction.

### Protein A mediates interaction with plasma purified C4BP

**Fig. 2.**
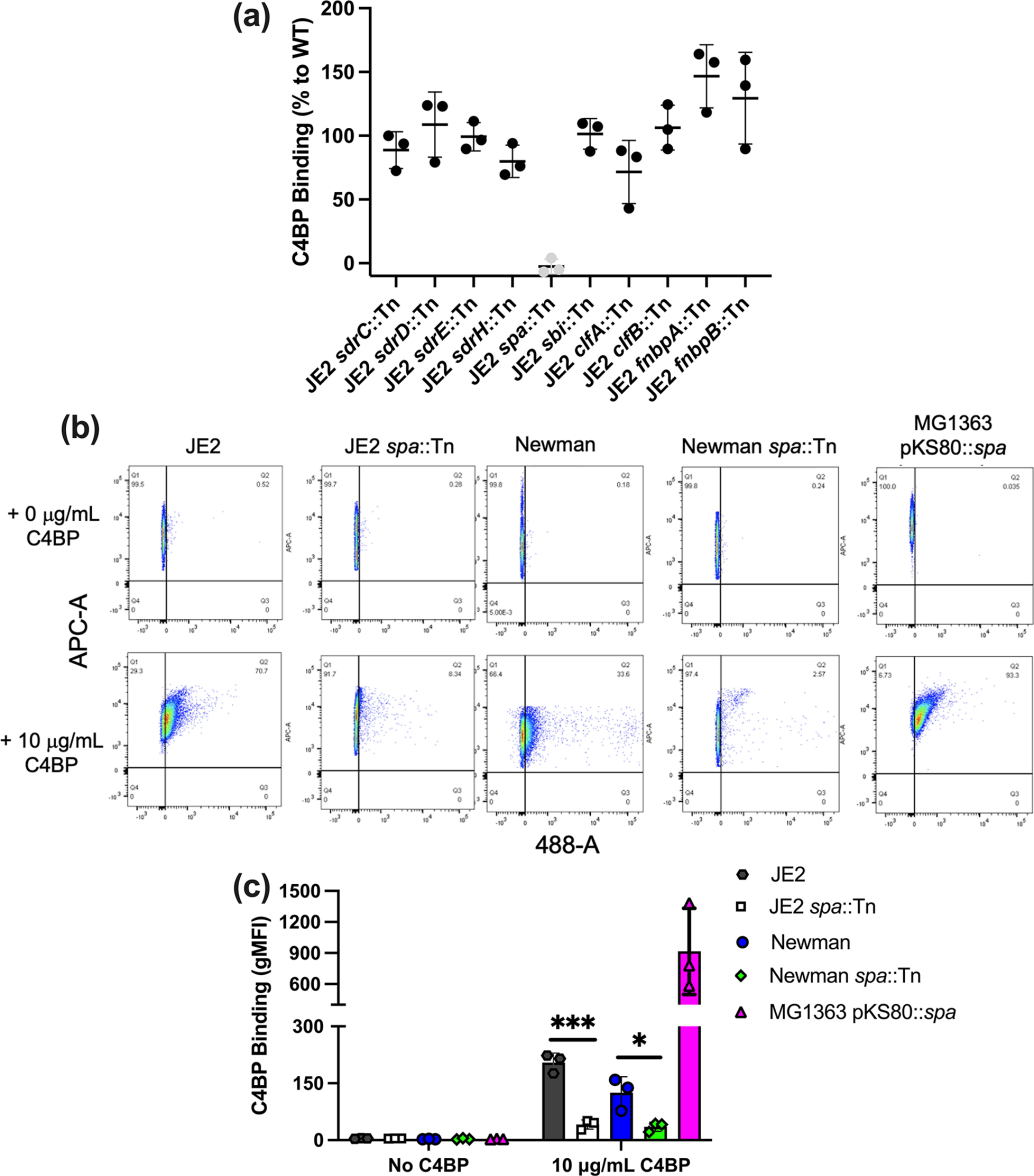
Protein A mediates interaction with plasma purified C4BP. (**a**) Illustrates the C4BP binding capacity of multiple cell wall-anchored protein transposon mutants (SdrC, SdrD, SdrE, protein A, ClfA, ClfB, FnbpA, and FnbpB) and Sbi, a non-covalently associated protein that is related to protein A. (b), (c) C4BP binding to *S. aureus* WT and transposon mutants of protein A and *L. lactis* expressing protein A. C4BP binding was analysed by measuring the geometric mean fluorescence intensity (gMFI) using a BD FACS Canto flow cytometer. Bars indicate the mean; data points represent three biological replicates and error bars inform the standard deviation. Statistical differences were calculated using a one-way ANOVA using Dunnett’s multiple comparisons test analysis comparing mutants to respective isogenic WT control. *P<0.05, ***P<0.001,.

To assess the importance of other CWA proteins in binding C4BP, we screened defined surface proteins [SdrC, SdrD, SdrH, protein A, Staphylococcus aureus binder of immunoglobulin protein (Sbi), clumping factor A (ClfA), ClfB, fibronectin binding protein A (FnBPA) and FnBPB] known to interact with serum and/or plasma proteins [21] (Fig. 2a). Interestingly, only the protein A mutant (*spa*::Tn) showed a significant decrease in C4BP binding in both JE2 and Newman backgrounds and similar to the no C4BP control (Fig. 2b, c). Overexpression of the gene encoding protein A (*spa*) in *L. lactis* resulted in binding of C4BP. The presence of Spa in the cell wall was confirmed using Western blotting (Fig. S2B). These results indicated that protein A played a major role in the recruitment of plasma-purified C4BP to the staphylococcal surface.

### C4BP is not recruited to *S. aureus* when incubated in human serum

**Fig. 3.**
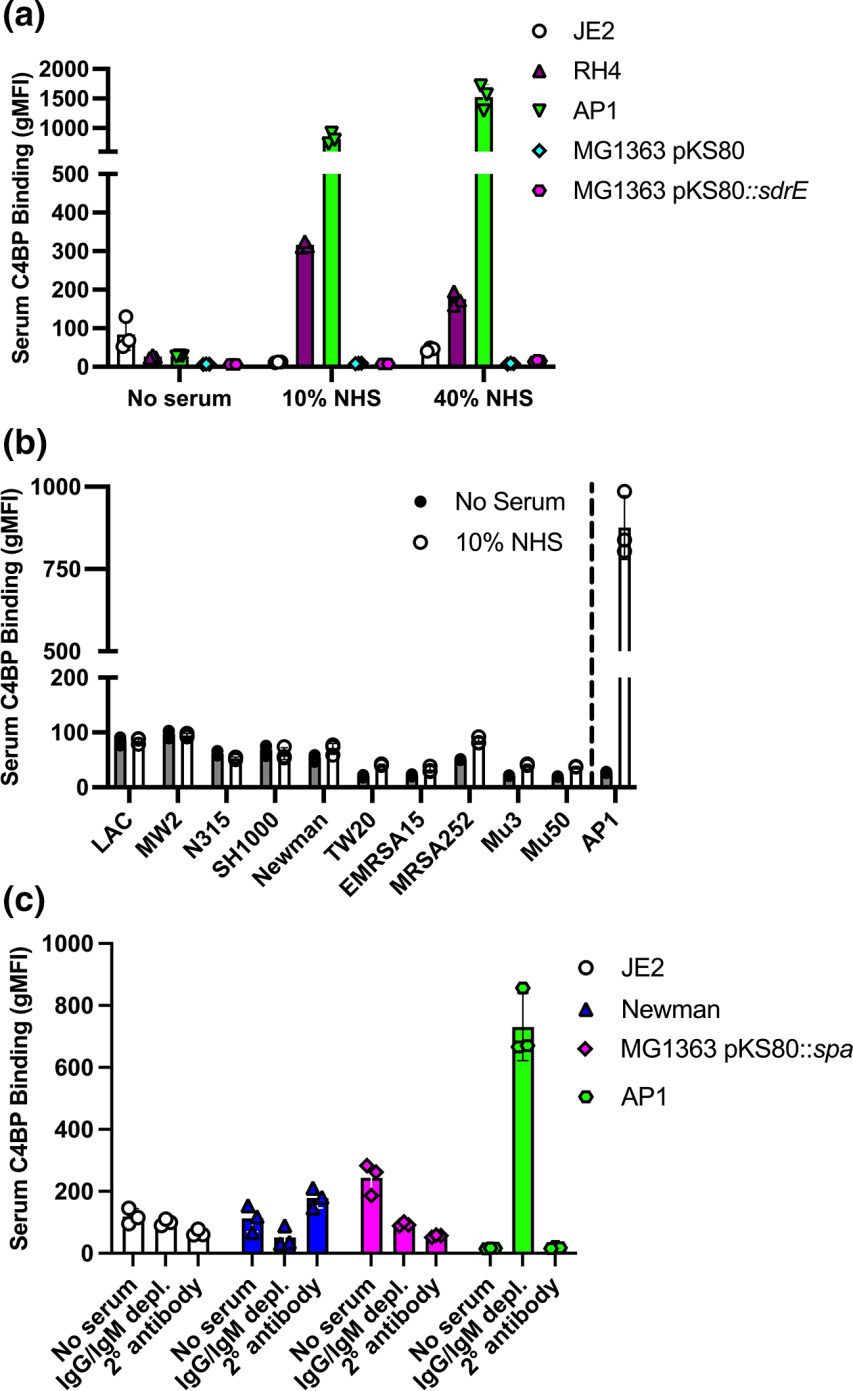
*
S. aureus* does not recruit C4BP when incubated in human serum. (a) C4BP recruitment to the bacterial surface was analysed following incubation of bacteria [* S. aureus* (JE2), *M. catarrhalis* (RH4), *S. pyogenes* (AP1) and *L. lactis* MG1363 with pKS80 empty control or pKS80 expressing SdrE] in either 10 % or 40% normal human serum (NHS). (b) Ten different *S. aureus* reference strains [LAC (clonal complex, CC))8; MW2 (CC1); N315 (CC5); SH1000 (CC8); Newman (CC8); TW20 (CC239); EMRSA15 (CC22); MRSA252 (CC30); Mu3 (CC5); Mu50 (CC5)] were examined for serum C4BP binding in 10 % NHS. All strains tested except SH1000 carry the *sdrE* gene. *S. pyogenes* strain AP1 is shown as a reference for a known C4BP binder. (c) C4BP recruitment was assessed using IgM/IgG depleted serum and compared to no serum and secondary antibody only controls. (a–c) Bound C4BP was detected using F(ab’)2 murine anti-human C4BP MK104 and goat anti-mouse AF-488 secondary antibody. The geometric mean fluorescence intensity (gMFI) was measured using a BD FACS Canto flow cytometer. Bars indicate the mean; data points represent three biological replicates and error bars inform the standard deviation.

Our results indicated that protein A was responsible for mediating the recruitment of C4BP to the staphylococcal surface. We next investigated C4BP binding from human serum using WT *S. aureus* strain JE2 and *L. lactis* expressing protein A (Fig. 3a) . To limit protein A mediated non-specific binding, we generated F(ab)’2 antibodies of the murine anti-human C4BP antibody MK104. Importantly, we could not observe C4BP binding from human serum using JE2 or *L. lactis* expressing protein A (Fig. 3a) . As additional controls we used previously characterised C4BP binders *Streptococcus pyogenes* strain AP1 and *Moraxella catarrhalis* strain RH4, which showed significant binding in line with previous data [22, 23] (Fig. 3a). In addition, we screened ten different *S. aureus* reference strains representing six different clonal complex types for serum C4BP binding. Again, we did not observe significant C4BP binding between serum and non-serum control (Fig. 3b). To investigate further we examined C4BP binding from IgM/IgG depleted serum as antibodies in serum may have prevented C4BP interaction with protein A, however we observed no C4BP interaction using depleted serum but did observe significant C4BP binding using *S. pyogenes* strain AP1 (Fig. 3c).

### Plasma purified C4BP interaction with *S. aureus* occurred due to IgG co-purified with C4BP

**Fig. 4.**
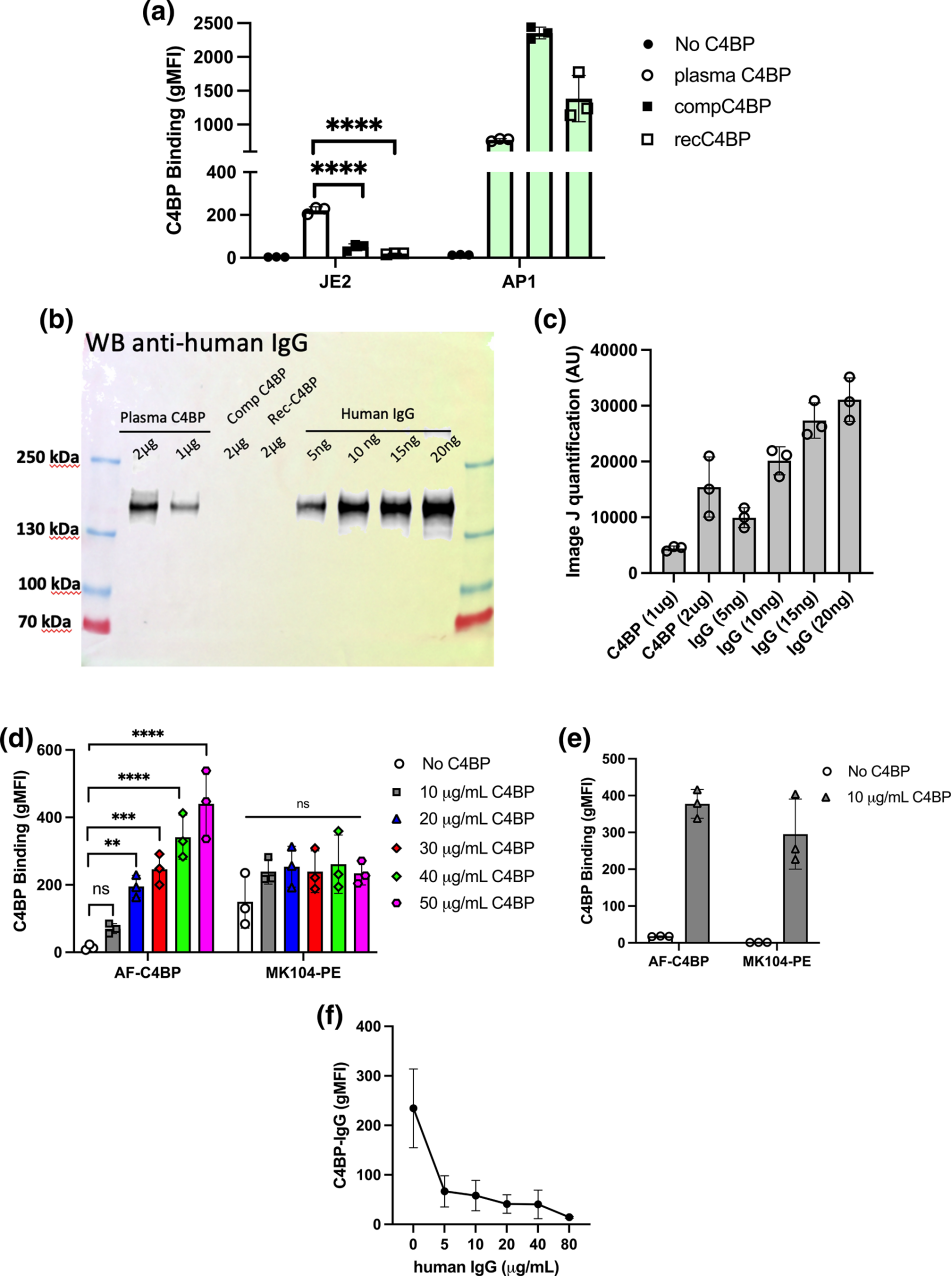
Plasma purified C4BP interaction with *S. aureus* occurred via IgG co-purified with C4BP. (**a**) Dy488-labelled plasma purified C4BP (plasma C4BP), commercially obtained C4BP (compC4BP) and recombinantly produced C4BP (recC4BP) were incubated with *S. aureus* strain JE2 or *S. pyogenes* strain AP1. Binding was analysed by measuring the geometric mean fluorescence intensity (gMFI) using a BD FACS Canto flow cytometer. (**b**) Plasma C4BP (1 and 2 µg), compC4BP (2 µg) and recC4BP (2 µg) were probed for human IgG contamination using goat anti-human IgG-HRP. Purified human IgG at concentrations between 5–20 ng was used as a control C) Image J quantification of western blot analysis (*n*=3; Fig. S2). (**d**) Dy488-labelled plasma C4BP was incubated with *S. aureus* strain JE2 at concentrations between 0–50 µg ml^−1^. Fluorescence was either measured at 488 or following incubation of bacteria with F(ab’)2 MK104 mouse anti-C4BP antibodies and secondary goat anti-mouse – PE antibodies. (**e**) Dy488-labelled plasma C4BP (10 µg ml^−1^) was incubated with *S. pyogenes* strain AP1 and binding analysed as described in (d). (**f**) *S. aureus* strain JE2 was pre-incubated with increasing concentrations of human IgG. Dy488-labelled plasma purified C4BP (10 µg ml^−1^) binding was analysed by measuring the gMFI using a BD FACS Canto flow cytometer. Bars indicate the mean; data points represent three biological replicates and error bars inform the standard deviation. (**a–d**) Statistical differences were calculated using a one-way ANOVA analysis using Dunnett’s multiple comparisons test. **P<0.01, ***P<0.001 ****P<0.0001,.

To understand why *S. aureus* could bind plasma purified C4BP but not C4BP present in human serum, we tested two other sources of C4BP; we used recombinantly produced C4BP (recC4BP) expressed and purified from human kidney 293 cells and C4BP obtained commercially (compC4BP). Both proteins were Dy-488 fluorescently labelled and the ability of *S. aureus* to bind to them was monitored using flow cytometry. Interestingly, *S. aureus* had significantly reduced binding to both recC4BP and compC4BP (Fig. 4a) compared to plasma C4BP preparation. As protein A was identified as the main staphylococcal C4BP binding protein, we tested if during preparation C4BP was in complex or co-purified with another protein A binding molecule, which led us to investigate IgG contamination. Using Western blotting and antibodies against human IgG we determined that our plasma purified C4BP was contaminated with human IgG whereas recombinantly produced and commercially obtained C4BP contained no traces of IgG (Figs 4b-c and S3). Image J quantification of anti-human IgG bands detected from our plasma purified C4BP compared to purified human IgG indicates 1 µg C4BP contains between 2.5–5 ng human IgG. To determine if IgG could form a complex with C4BP and mediate the recruitment of C4BP to the bacterial surface we determined the presence of labelled plasma purified C4BP on *S. aureus* using F(ab’)2 MK104 antibodies and a secondary antibody labelled with a non-overlapping phycoerythrin fluorophore (Fig. 4d). Increasing concentrations of Dy-488 labelled C4BP resulted in increased 488 fluorescence in a dose-dependent manner, however no increase in C4BP binding using the MK104-PE combination was observed and no statistically significant difference was observed between no C4BP control and 10–50 μg ml^−1^ C4BP. These results confirm that *S. aureus* was interacting solely with fluorophore labelled IgG that was co-purified with C4BP during plasma C4BP purification. As a control we tested *S. pyogenes* AP1 binding to 10 µg ml^−1^ Dy-488 labelled C4BP and observed binding both via Dy-488 and PE gating (Fig. 4e), indicating the presence of C4BP on the streptococcal surface in line with previous results.

Lastly, pre-incubation of *S. aureus* strain JE2 with human IgG prevented binding of IgG contaminated C4BP (C4BP-IgG) indicating that protein A mediated binding of C4BP-IgG was via protein A domains that recruit human IgG (Fig. 4f).

### Pre-incubation of *S. aureus* with C4BP had no effect in limiting complement deposition

**Fig. 5.**
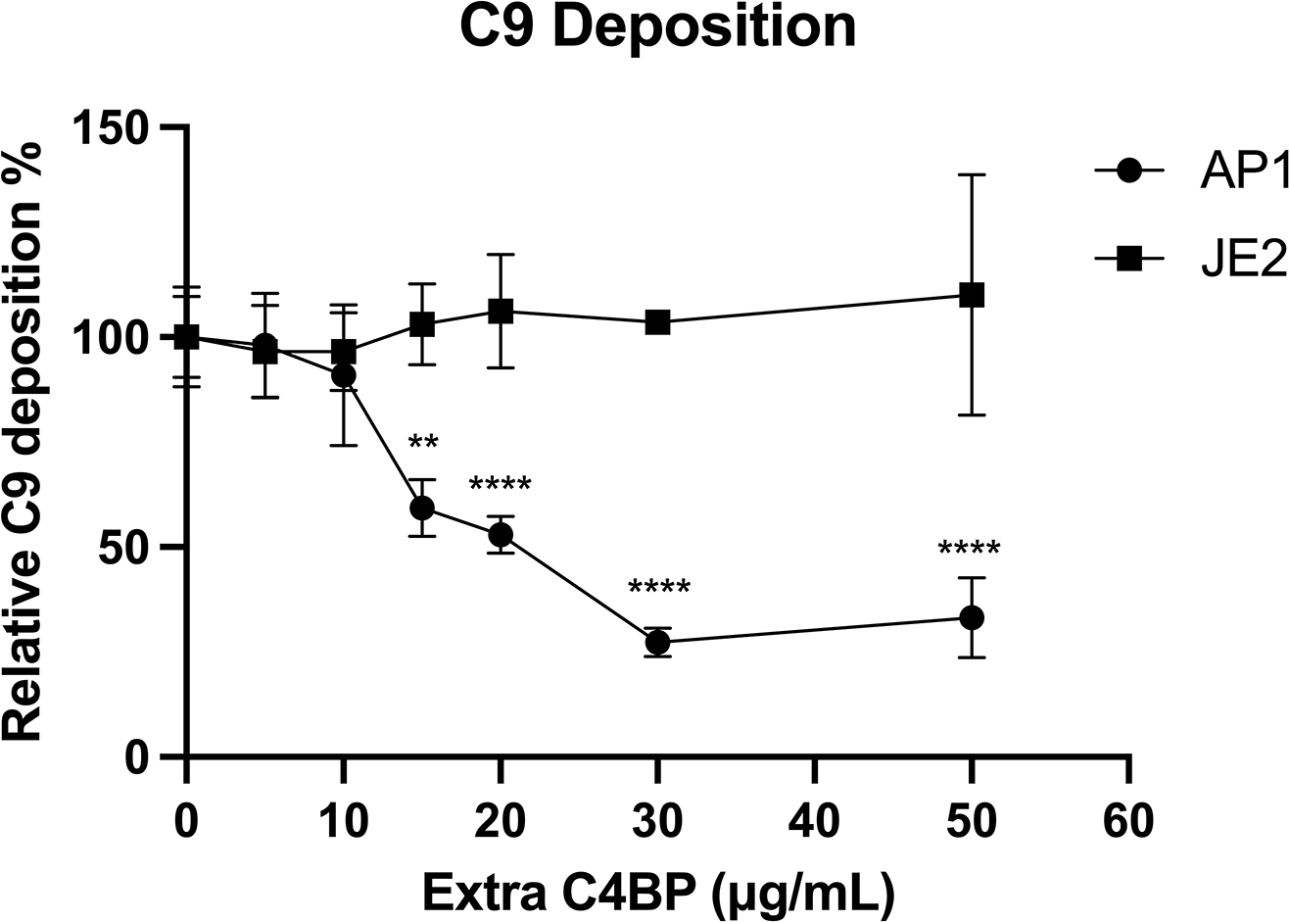
Pre-incubation of *S. aureus* with C4BP had no effect in limiting complement deposition. *S. aureus* strain JE2 or *S. pyogenes* strain AP1 were pre-incubated with increasing concentrations of C4BP prior to incubation in 8 % NHS. C9 deposition was analysed using goat anti-human C9 antibody and rabbit-anti-goat AF-488 secondary antibody with the geometric mean fluorescence intensity (gMFI) using a BD FACS Canto flow cytometer. Symbols represent the mean of three biological replicates and error bars inform the standard deviation. Statistical differences were calculated using a two-way ANOVA using Sidak’s multiple comparison test. **P<0.01, ****P<0.0001,.

We tested the outcome of pre-incubating *S. aureus* or *S. pyogenes* with increasing concentrations of C4BP (containing no bound IgG) on complement deposition. *S. pyogenes* incubated with exogenously added C4BP concentrations above 15 µg ml^−1^ in human serum resulted in a statistically significant reduction of C9 deposition compared to *S. aureus* JE2 (Fig. 5). This data shows that C4BP was functional in reducing complement deposition on *S. pyogenes* when the serum was supplied. Noteworthy, under the same conditions we observed no difference in the level of C9 deposition on *S. aureus* when pre-incubated with concentrations of up to 50 µg ml^−1^ C4BP (Fig. 5).

## Discussion

*S. aureus* is a master of immune evasion, expressing multiple proteins that interfere with complement activity, antibody recognition, and immune cell recruitment, activation and killing mechanisms [24]. In this study we investigated whether *S. aureus *, like other human pathogens, utilizes the recruitment of the soluble complement regulator C4BP as an immune evasive defence mechanism. Initially our results indicated that *S. aureus* bound C4BP via protein A and not through the previously identified SdrE protein. However, we could not observe any C4BP binding from serum using a panel of diverse *S. aureus* strains but did observe significant binding of C4BP by pathogenic bacteria, *S. pyogenes* and *M. catarrhalis *, that have been previously identified as C4BP binders [22, 23], validating our flow cytometric, bacteria-protein binding experiments.

Protein A is a highly abundant cell-wall-anchored protein expressed by the vast majority of *S. aureus* isolates [25]. Protein A can capture IgG molecules via the Fc region, interrupting antibody-mediated phagocytosis [26] and preventing IgG hexamerization required for classical complement pathway activation [27]. We tested the ability of *S. aureus* to bind to recombinantly produced (rec-C4BP) and commercially purchased (compC4BP) C4BP, however we observed no interaction with *S. aureus *. This indicated that plasma purified C4BP may contain contaminants that have been fluorescently labelled along with the purified C4BP and are recognized by protein A. We tested the three sources of C4BP and found that plasma purified C4BP contained minute quantities of human IgG whereas recC4BP and compC4BP contained no detectable IgG. Previous work has shown that C4BP has several different binding partners including plasma, ECM and amyloid proteins [6], therefore we hypothesized that C4BP may form complexes with IgG in serum and *S. aureus* could recruit C4BP to the surface via interaction with bound IgG. Using antibodies that recognize labelled plasma purified C4BP we were able to determine that C4BP was not localized to the staphylococcal surface, and *S. aureus* was interacting solely with IgG that was co purified during C4BP purification and Dy-488 labelled along with C4BP. C4BP interacts with C4b which can be bound to immune complexes, therefore during C4BP purification very minor contamination of plasma purified C4BP with IgG/IgM and C4b may occur as observed in this study where IgG was detected at 2.5–5 ng per μg of C4BP. Combined, our results indicate that *S. aureus* does not recruit C4BP to the bacterial surface. Importantly, analysis of the cell surface proteome of *S. aureus* and its interaction with human serum proteins did not identify C4BP on the cell surface of *S. aureus* strain LAC or Newman [28].

Previous experiments to determine C4BP recruitment to the staphylococcal surface incubated *S. aureus* in human serum and then employed 2 % SDS to strip bacterial cells of surface proteins, determining the presence of C4BP through dot blot using monoclonal anti-C4BP antibodies [10, 11]. Using this approach, it was shown that significant C4BP binding was observed on the non-pathogenic *L. lactis* empty vector control, with binding of greater than 1 µg ml^−1^ C4BP reported [10]. This is surprising as to date, all confirmed C4BP binders are pathogenic microorganisms [8]; experiments that investigated the ability of endogenous microflora to recruit complement inhibitors showed no interaction [29], however, a thorough investigation using diverse microflora has not been reported. In addition, experiments using purified C4BP were not tested for IgG contamination and dot blot analysis was not carried out using F(ab)’2 generated antibodies and therefore the results may be distorted by the presence of protein A in the stripped cell extracts. Furthermore, human serum proteins have a high propensity for binding polymers such as polypropylene and polystyrene [28, 30], which form the basis of standard microcentrifuge tubes and microplates. Adsorption of proteins onto polymers is predominantly mediated via hydrophobic interactions [31]. Therefore, it is feasible that during the incubation of *S. aureus* with NHS, human serum proteins including C4BP can bind to the reaction vessels and remain following the washing stages. Treatment with SDS during the cell-stripping stage would release proteins including C4BP from the reaction vessel producing erroneous dot blot results informing that *S. aureus* binds C4BP. In contrast, our analysis described here uses highly sensitive flow cytometry of intact bacterial cells to determine C4BP recruitment, which would limit any binding artefacts that may arise following treatment with SDS and only report on bound protein on the bacterial surface.

Gaining a complete understanding of the intricate methods used by pathogens to manipulate immune responses is crucial for the design of future immunotherapeutics. Confirming whether microbial pathogens recruit soluble complement inhibitors is central for the development of chimeric fusion proteins designed to simultaneously disrupt the recruitment of complement inhibitors while activating complement on microbial surfaces, as we and others have recently developed as novel anti-infective immunotherapeutics [32–34]. Work presented in this study offers new insights into complement evasion strategies employed by *S. aureus* and may help design future intervention strategies to combat this human pathogen.

## Supplementary Data

Supplementary material 1
