# Supplementary material for: Recruitment of C4b-binding protein is not a complement evasion strategy employed by Staphylococcus aureus
Source: Microbiology (Reading). 2023 Sep 5;169(9):001391. doi: 10.1099/mic.0.001391 (PMC10569063; doi:10.1099/mic.0.001391)
Supplement: Supplementary material 1 [file mic-169-1391-s001.pdf]

Supplementary Material

Recruitment of C4b-binding protein is not a complement evasion strategy employed by *Staphylococcus aureus*

Shuxian Li[1], Serena Bettoni[2], Frida Mohlin[2], Joan A. Geoghegan[3], Anna M. Blom[2] and Maisem Laabei[1]*.

1: Department of Life Sciences, University of Bath, Bath, BA2 7AY, United Kingdom
2: Division of Medical Protein Chemistry, Department of Translational Medicine, Lund University, Malmö, Sweden.
3: Institute of Microbiology and Infection, University of Birmingham, Edgbaston, Birmingham, B15 2TT, United Kingdom

For correspondence, please email ml418@bath.ac.uk

Keywords: *Staphylococcus aureus,* immune evasion, complement, C4b-binding protein

Materials and Methods

Western immunoblot of bacterial surface proteins

L. lactis strains were grown O/N in M17 broth with 1% glucose. Bacterial cells were pelleted and normalised to OD600=40 in digestion buffer (50 mM Tris-HCL, 20 mM $MgCl_2$, 30% [w/v] raffinose, pH7.5) containing protease inhibitor cocktail Set VII (Merck). Cell wall proteins were solubilized by digestion with lysozyme for *L. lactis* (400 µg/mL) at 37°C for 1 h. Protoplasts were harvested by centrifugation (7,000 x *g*, 15 min), and 20 µL supernatants were boiled in sample buffer and subjected to SDS-PAGE using mPAGE™ Bis-Tris 4-12% Precast Gels (Merck). SDS-PAGE gels electro-transferred onto nitrocellulose membranes, blocked with 5% skimmed milk and probed with either swine anti-rabbit HRP (1:2000, abcam) for Spa or rabbit anti-SdrE serum (1:1000) for SdrE. Rabbit anti-SdrE were detected by swine anti-rabbit- HRP and bound antibody were visualised using Amersham ECL start western blotting detection reagent (Cytiva).

Supplementary Figures:

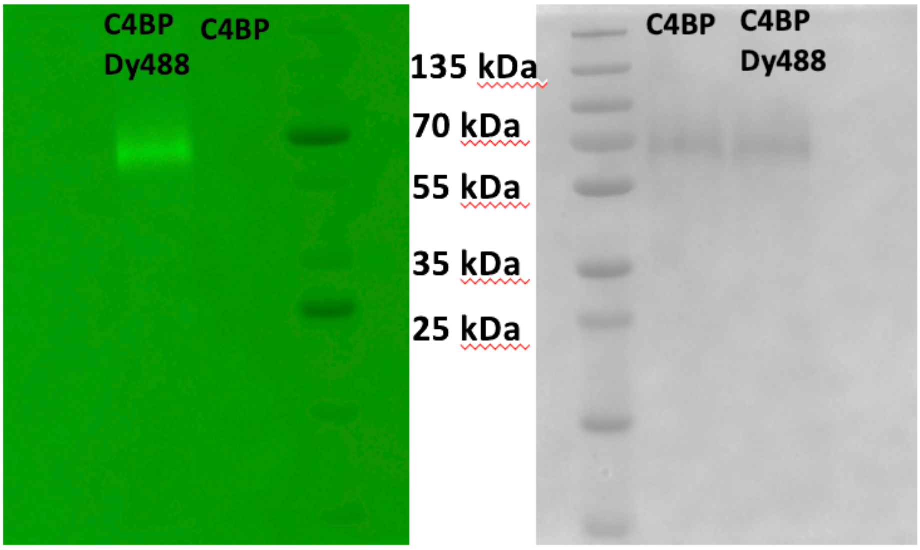

Supplementary Figure 1: Dy488-labelled plasma purified C4BP. Plasma purified C4BP was labelled with DyLight-488 as described in Methods. SDS-PAGE (10% w/v acrylamide / bisacrylamide) was loaded with 3 µg of either labelled or unlabelled C4BP. The reduced samples were imaged using a fluorescent gel imaging system (Azure Biosystems) or stained with Coomassie brilliant blue revealing a protein band corresponding to the α-chain of C4BP (~70 kDa).

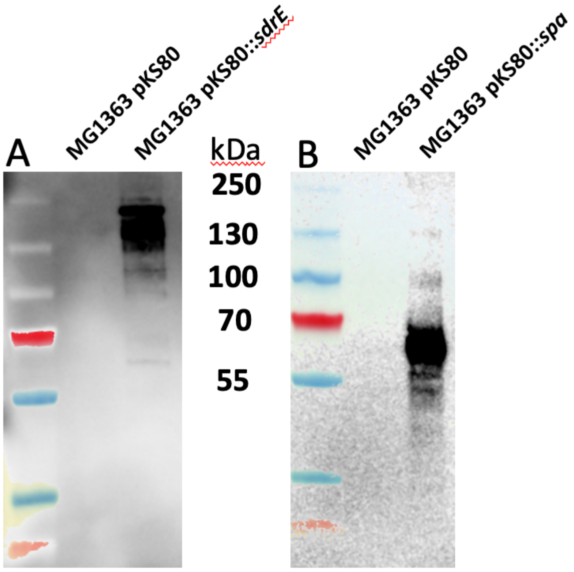

Supplementary Figure 2: Expression of SdrE and Spa in *S. aureus* JE2 and *L. lactis* MG1363. Surface proteins of *S. aureus* strain JE2 or *L. lactis* strain MG1363 harbouring either empty expression vector pKS80, pKS80 expressing SdrE or pKS80 expressing Spa were extracted as described in the Materials and Methods. **A)** SdrE expression was confirmed using rabbit anti-SdrE serum and swine anti-rabbit HRP secondary antibody. SdrE expression is noted as a band at ~180kDa **B)** Spa expression was confirmed using swine anti-rabbit IgG-HRP, noted as a band at ~60kDa.

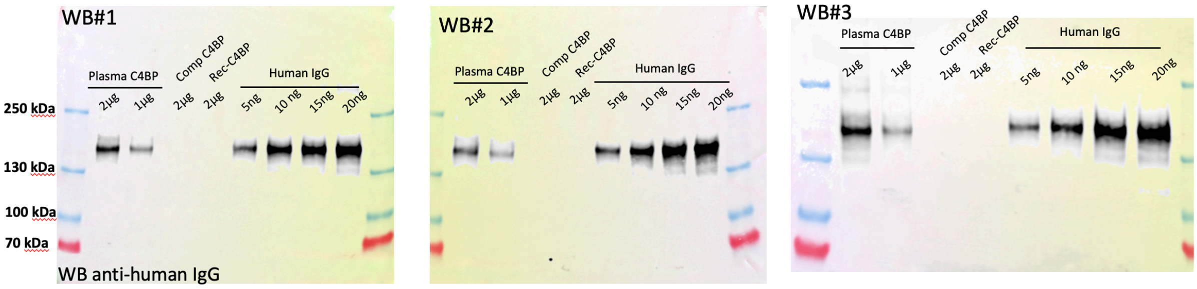

Supplementary Figure 3: IgG contamination of C4BP measured using western blot. Plasma C4BP (1 and 2 µg), compC4BP (2 µg), recC4BP (2 µg) and purified human IgG at concentrations between 5-20 ng were subjected to a non-reducing SDS-PAGE (5% w/v acrylamide / bisacrylamide) and probed for human IgG contamination using goat anti-human IgG-HRP revealing a band corresponding to non-reduced human IgG (~150 kDa). Western blot analysis was performed in triplicate.
